# Experimental Studies on the Seismic Performance of Prefabricated Circular Hollow Bridge Piers Constructed with PVA Fiber Concrete

**DOI:** 10.3390/ma16051981

**Published:** 2023-02-28

**Authors:** Jun Shi, Yuang Deng, Yi Zhang, Feiting Shi, Jian Yang

**Affiliations:** 1 School of Civil Engineering, Central South University, Changsha 410075, China; 2 National Engineering Laboratory for High-Speed Railway Construction, Changsha 410075, China; 3 Academy of Combat Support, Rocket Force University of Engineering, Xi’an 710025, China; 4 School of Civil Engineering, Yancheng Institute of Technology, Yancheng 224051, China

**Keywords:** seismic performance, PVA concrete, prefabricated hollow piers, shear strength, quasi-static test

## Abstract

To investigate the seismic performance of prefabricated circular hollow piers with socket and slot connection, eight 1/3.5-scale specimens constructed with polyvinyl alcohol (PVA) fiber at the pier body were tested. The main test variables included the axial compression ratio, grade of pier concrete, shear-span ratio, and stirrup ratio. The seismic performance of prefabricated circular hollow piers was studied and analyzed from the aspects of the failure phenomenon, hysteresis curve, bearing capacity, ductility index, and energy dissipation capacity. The test and analysis results showed that all specimens suffered from flexural shear failure, and the increase in axial compression ratio and stirrup ratio would lead to more significant spalling of the concrete at the bottom of the specimen, but the existence of PVA fiber would improve this phenomenon. In a certain range, the increase in axial compression ratio, stirrup ratio, and the decrease in shear span ratio can improve the bearing capacity of the specimens. However, an excessive axial compression ratio would easily lead to a decrease in the ductility of the specimens. The increase in the stirrup ratio and shear-span ratio caused by the change in height can improve the energy dissipation characteristics of the specimen. On this basis, an effective shear-bearing capacity model of the plastic hinge area of prefabricated circular hollow piers was proposed, and the prediction effects of specific shear capacity models on test specimens were compared.

## 1. Introduction

In recent years, with the rapid development of highway construction around the world, prefabricated bridge piers have been widely used in urban viaducts and sea-crossing bridges. This kind of prefabricated structure has the advantages of high construction efficiency and stable quality control [1,2]. In addition, its construction has little impact on the surrounding traffic and natural environment [3,4]. Columns are the most critical load-carrying elements of bridge structures [5], and thus, for some cities or regions with frequent earthquakes, the seismic performance and resilience of prefabricated piers has become one of the most significant factors for people to consider [6,7,8,9].

The seismic performance of prefabricated piers is closely related to the connection form between different prefabricated components, which has been partially studied by many scholars. Ameli et al. [10,11] studied the response of prefabricated specimens and cast-in-place specimens with different forms of joints at the joints under cyclic quasi-static loads and analyzed their hysteretic energy dissipation properties. Lu et al. [12] compared the failure mode, energy dissipation capacity, and stiffness degradation performance of prefabricated columns and cast-in-place columns connected by vertical grouting collars through experiments. Han et al. [13,14] conducted model tests and finite element analysis on plug-and-socket joint piers and studied the influence of insertion depth on the seismic performance of such piers. Liu [15] designed a scaled model test to study the positioning effect of the grouting sleeve connection and its advantages and disadvantages with the seismic performance of cast-in-place column specimens. Fu [2] compared and analyzed the seismic performance of cast-in-place square piers, precast solid square piers, and precast square piers with hollow sandwiches through the quasi-static test, and Fu [16] also studied the seismic performance of prefabricated steel tube-confined concrete circular pier with grouting sleeve connection using the Finite Element Method. It can be seen that most researchers focus on the influence of connection type-related parameters on the seismic performance of prefabricated piers, and other researchers are concerned with the comparison between the performance of prefabricated piers and cast-in-place piers [17,18]. However, the study on the influence of some basic factors on the seismic performance of prefabricated piers is not comprehensive enough. Therefore, it is worthy of further study in this aspect.

In addition, the materials used and the section form of the pier also have an important influence on the seismic performance and bearing capacity of pier [19,20,21]. Han et al. [22] found that polyvinyl alcohol fiber reinforced concrete (PVA-FRC) columns have better ductility, energy dissipation capacity and seismic performance than ordinary reinforced concrete columns. Su et al. [23] studied four PVA fiber-reinforced ultra-high performance concrete short columns with a solid rectangular section, and he found that PVA fiber could improve the ultimate elastic-plastic displacement angle and energy dissipation capacity of piers. Cassese et al. [24] studied the shear failure modes of four hollow rectangular section piers under cyclic loading and compared them with existing literature and models. According to the literature statistics, among studies that adopted the cyclic loading pattern to investigate the behavior of hollow RC columns, only a tiny part of studies focused on shear strength [25,26]. In general, there are few studies on the shear models of the plastic hinge region of hollow pier columns, and the applicability of the existing models for piers with different section combinations is not clear. More test data of specimens are needed for verification.

Based on the above situation, eight prefabricated circular hollow pier specimens with PVA fiber concrete were designed in this paper. According to the results of quasi-static tests, the influence rules of axial compression ratio, grade of pier body concrete, shear span ratio, and stirrup ratio were studied, and the failure behavior, hysteresis energy dissipation, and bearing capacity of the specimens under the above-mentioned factors were analyzed. In addition, combined with the analysis results of the test data and the existing specifications, the shear bearing capacity formula suitable for the plastic hinge region of the prefabricated hollow circular piers was fitted. In addition, the applicability of the existing shear models for predicting the bearing capacity of such structures was compared. These studies have important reference value for the analysis of multi-factor parametric rules and structural seismic design of prefabricated hollow piers. In addition, the proposed shear capacity model can predict the failure load of this kind of structure effectively and safely.

## 2. Experimental Program and Setup

### 2.1. Model Design and Material Properties

**Figure 1:**
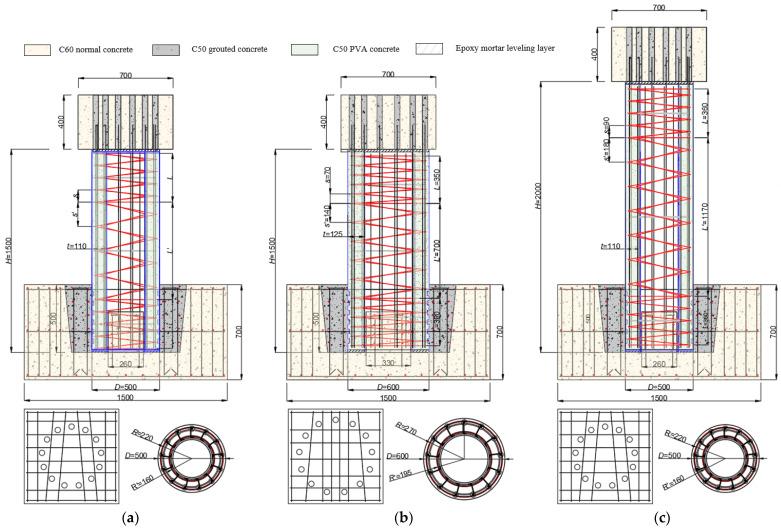
The size and reinforcement layout of specimens (mm), (**a**) Type I: S1~S4, S7 and S8 (**b**) Type II: S5 (**c**) Type III: S6.

In terms of the equipment conditions of the laboratory and the factors to be studied, a total of eight 1/3.5-scale prefabricated circular hollow piers were designed in this experiment, each composed of a cap beam, pier body, and foundation. The pier body and foundation were connected by slot type: a positioning block was arranged inside the precast foundation for the hollow pier body to be inserted, then the two parts were integrated by pouring fine stone concrete into the remaining section. While the socket connection was used between the pier body and cap beam: the corrugated steel pipes were embedded in the prefabricated cover beam for the longitudinal reinforcement extending out of the pier body to be inserted. Then, the two parts were integrated by pouring grouting material into the steel pipes. The dimensions of the foundation and the cover beam of all specimens were 1.5 m × 1.5 m × 0.7 m and 0.7 m × 0.7 m × 0.4 m, respectively. According to the pier body height and section size, eight specimens can be divided into three types, as shown in Figure 1. The pier contains two reinforcement cages. The longitudinal reinforcement is of HRB400 grade while the low carbon steel wires, whose tensile strength is 360 MPa, were used as the spiral stirrup and the rest tie bars are of HPB300 grade. More specific dimensions related to the specimens and the internal reinforcement layout are shown in Figure 1. Table 1 summarizes the critical design parameters and test variables of specimens. Specimen S2 was used as the control group, and specimens S1 and S3 were used to study the effects of axial compression ratio (*n* = 0.05 and 0.15) on the seismic performance of prefabricated circular hollow piers. The S4 specimen was designed to study the influence of the change in pier concrete’s grade (C50), and the specimens S5 and S6 were designed to study the influence of the change in shear-span ratio (λ = 2.0 and 3.4) caused by changing the pier’s diameter and height, respectively. For specimens S7 and S8, the diameter and spacing of the stirrup were changed to study the effect caused by the stirrup ratio (*ρ_sv_* = 0.34 and 0.91).

**Figure 2:**
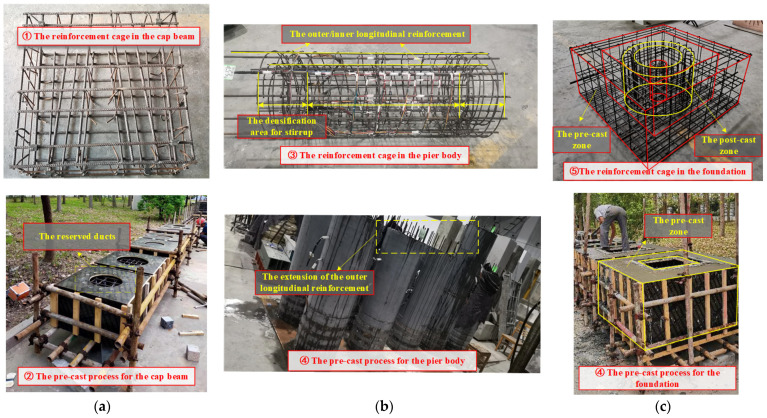
The process of fabrication for different parts, (**a**) Part I: cap beam (**b**) Part II: pier body (**c**) Part III: foundation.

**Figure 3:**
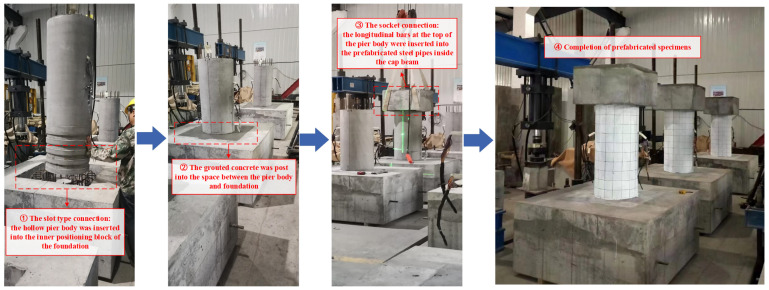
The main process of assembly.

According to the strength (C60) of normal concrete pier in actual engineering prototype and the completed test results [27], two different grades of PVA concrete were prepared in this paper. The PVA fibers used are bundled monofilament with a diameter of 15 μm and a length of 10 mm, purchased from the Kuraray Company (Shanghai, China) according to the preliminary material performance test [27], and its tensile strength is about 1400 Mpa. The concrete mix of the pier body is shown in Table 2. The compressive strength of different masonry materials listed in Figure 1 has been measured, and the results are shown in Table 3. After completing the material configuration and basic mechanics tests, the eight designed specimens were fabricated and assembled according to the test plan. The primary process of fabrication and assembly is shown in Figure 2 and Figure 3.

### 2.2. Loading Setup and Test Instrument

**Figure 4:**
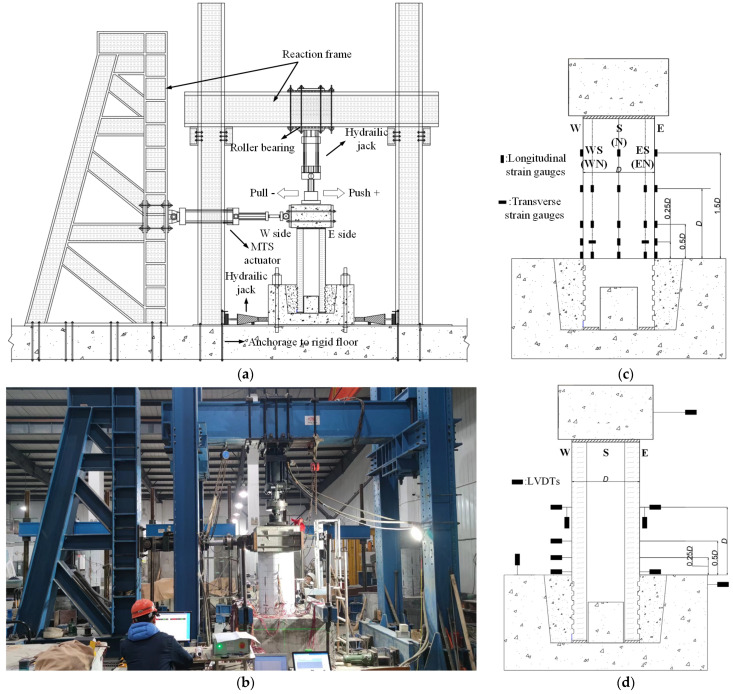
The layout of environment and equipment, (**a**) The loading equipment, (**c**) The layout of strain gauges, (**b**) The field environment, (**d**) The layout of LVDTs.

The whole process of fabrication and loading was carried out at Yancheng Institute of Technology (Jiangsu, China). Figure 4a,b show the quasi-static test’s field environment and equipment layout. The loading equipment of this test mainly includes the vertical loading device and the horizontal loading device. During the test, the vertical force was applied by a 3000 kN hydraulic jack. The jack was connected to the reaction beam by rotating the hinge to ensure that the jack always contacted the loading surface on the top of the cap beam during the loading process. The horizontal force was applied by a 1000 kN electro-hydraulic servo horizontal actuator, and the other end of the actuator was fixed on the reaction wall to provide low-cycle horizontal force for the specimen. In addition, two beams were set on the top surface of the foundation, and reaction frames were set on the side to ensure that the foundation of the specimen was rigidly connected to the ground during the test. The main parameters, including displacement, loading force, strain, and crack width, were measured in the test. Linear variable differential transducers (LVDTs) and electrical resistance strain gages were used to measure the displacement at critical locations along the pier’s height and monitor the specimen’s internal damage degree. Moreover, the specific layout position is shown in Figure 4c,d. During the test, the loading force imposed on the cap beam was collected by the electro-hydraulic servo testing machine collection system, the crack development trend of the pier was manually marked, and the crack width change was measured and recorded by the ZBL-F800 comprehensive crack tester.

### 2.3. Loading Protocol

**Figure 5:**
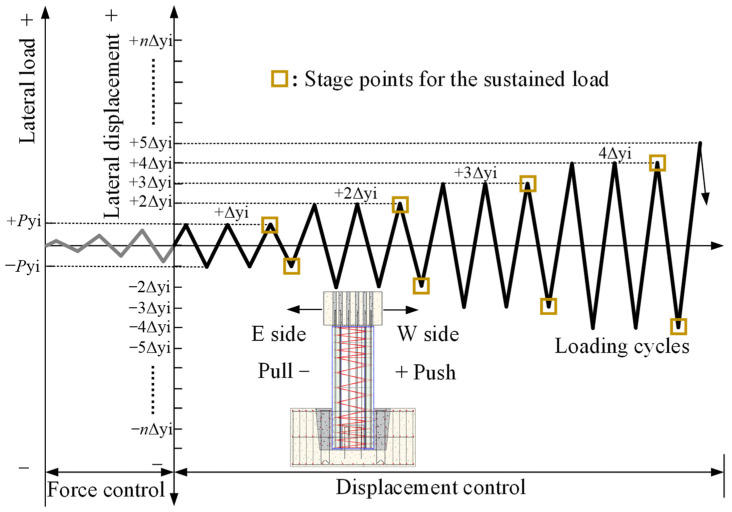
The loading sequence and rule of specimen.

The method of load-displacement mixed control loading was used in the test. Before the formal loading, a certain axial and horizontal load were applied to the specimen, and the working state of the specimen and device was verified by observing the change in strain value. Then, the pseudo-static test was conducted, and the loading sequence and rules of specimens are shown in Figure 5. First, the constant target axial pressure was applied to the specimen according to the designed axial compression ratio [28]. Then, the lateral load was gradually increased by force control mode according to the trial calculation results of the finite element model until the initial cracking of the specimen occurred. Subsequently, the displacement control mode was adopted, and the horizontal displacement (Δ*_yi_*) corresponding to the initial cracking of the specimen was taken as the primary displacement increment under the displacement loading mode. Each displacement level was repeated three times, and the load was held for 2 min when the displacement amplitude of the third cycle was loaded so that cracks in the pier body could fully develop and be observed. When the lateral bearing capacity of the specimen dropped to 85% of the maximum bearing capacity, the test loading was terminated.

## 3. Test Results and Discussion

### 3.1. General Behavior and Mode of Failure

**Figure 6:**
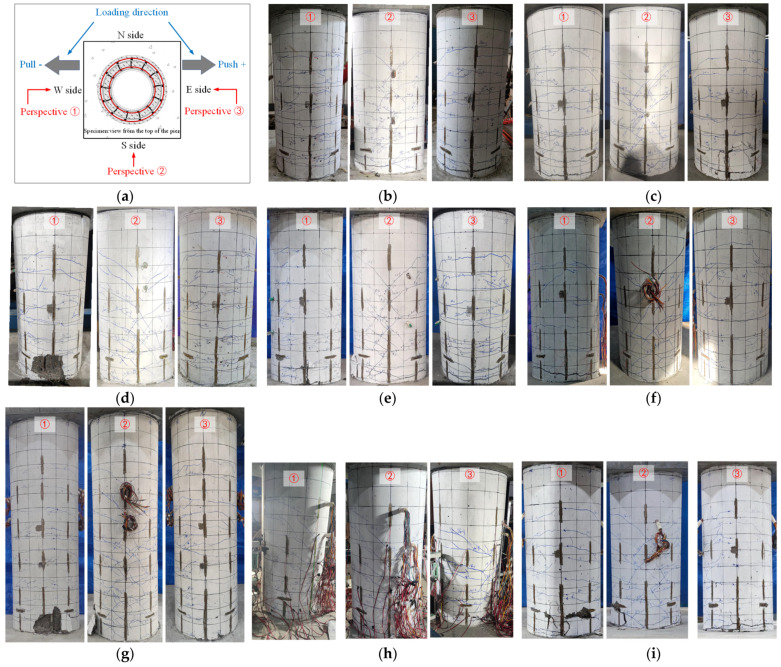
Failure phenomenon and mode of each specimen, (**a**) Different perspectives for observation, (**b**) S1, (**c**) S2, (**d**) S3, (**e**) S4, (**f**) S5, (**g**) S6, (**h**) S7, (**i**) S8.

Figure 6 shows the cracking morphology and typical failure process of eight specimens observed from three different perspectives after the test. It can be found that all specimens experienced the development and expansion of bending cracks and inclined cracks, as well as the spalling of concrete at the bottom of the pier body. At last, all specimens formed the plastic hinge as expected, eventually leading to the shear failure. The specific failure process of each specimen is described as follows. For specimens S1, S2, and S3 with different parameters of axial compression ratio (0.05, 0.10, and 0.15), no apparent cracks appeared when the lateral displacement was still small (<4 mm), and the loading and unloading curves of the specimens could overlap into a straight line. With the increase in loading displacement, specimen S1, with a lower axial compression ratio, appeared to have transverse cracks in the height range of 20 cm at the lower part of the pier earlier. Subsequently, the original transverse cracks of the three specimens continued to develop along the horizontal direction, and new transverse cracks began to appear along the height of the pier. At the same loading displacement level, the height of the cracks decreased with the increase in the axial compression ratio of the specimens. With further loading displacement, the existing and new transverse cracks began to cross diagonally downward in the south and north of the pier body. The longitudinal bars inside the specimen reached the yield strength. After the transverse load of the specimen reached the peak, the specimens entered the stage of bearing capacity degradation, and the crack on the piers began to turn to the width development. When specimens entered the failure stage, the pier of specimen S1 only partially peeled off on both sides of the loading direction, showing better ductility [29] while the PVA concrete at the bottom of the pier of specimens S2 and S3 showed more obvious swelling and peeling. Furthermore, the scope of the spalling of specimen S3 was more extensive, and the steel bars exposed reflected more obvious bending deformation.

Compared with specimen S2, the concrete pier grade of specimen S4 is lower, but the overall failure mode was basically the same. The difference is that under the same loading displacement of specimen S4, the initial horizontal cracks appeared earlier, and the oblique crack developed earlier. For specimen S5, with a larger pier diameter, compared with specimen S2, horizontal cracks appeared under smaller loading displacement. With the further increase in loading displacement, more dense cracks appeared within the height range of 60 cm from the pier bottom. Once the cracks on both sides of the pier body appeared, they quickly crossed in the middle. When the specimen entered the failure stage, it could be seen that the PVA fibers between the cracks, with the longitudinal bar inside the pier body, were pulled off. The main crack width of the lower part of the pier formed rapidly increased, and the specimen’s lateral bearing capacity decreased sharply. As for specimen S6, in the early loading stage, the distribution range and spacing of horizontal cracks on both sides of the pier body were more extensive, and the maximum height of transverse cracks from the pier bottom reached 80 cm at this stage. With the further increase in the loading displacement level, it can be found that more and more dense horizontal cracks appeared in the height range of 60 cm from the bottom of the pier. Then, the horizontal cracks continuously developed diagonally downward until the lateral load peaked. Subsequently, a peeling phenomenon appeared at the bottom of the pier in the broader area. The visible principal cracks formed gradually in the peeling place, leading to the overall spalling of concrete within the scope of the pier bottom. 

In terms of specimens S7 and S8 with a change in the stirrup ratio, the cracks’ formation and development characteristics were basically consistent in the initial stage of displacement control. As the loading displacement continued to increase, the bars inside specimen S7 yielded first and entered the bearing capacity degradation stage. At this stage, there were no new cracks in the pier along the height direction, but obvious principal cracks could be seen in the range of 10 cm from the bottom of the pier. As the crack width increased gradually, the surrounding concrete cracks appeared to be peeled until specimen S7 was damaged when the bearing capacity decreased to 85% of the peak load. Relatively, specimen S8 reached the peak bearing capacity at a more significant displacement loading level and entered the degradation stage. It can be found that a principal crack appeared within the range of 10–20 cm from the pier bottom. The principal cracks continued to develop along the pier body, and its width increased, forming a concrete spalling area with other principal cracks. At the same time, it could be heard that there was a dull sound of steel reinforcement being pulled off inside the pier body, and then the bearing capacity of specimen S8 decreased rapidly and the specimen was damaged.

### 3.2. Load–Displacement Behavior

**Figure 7:**
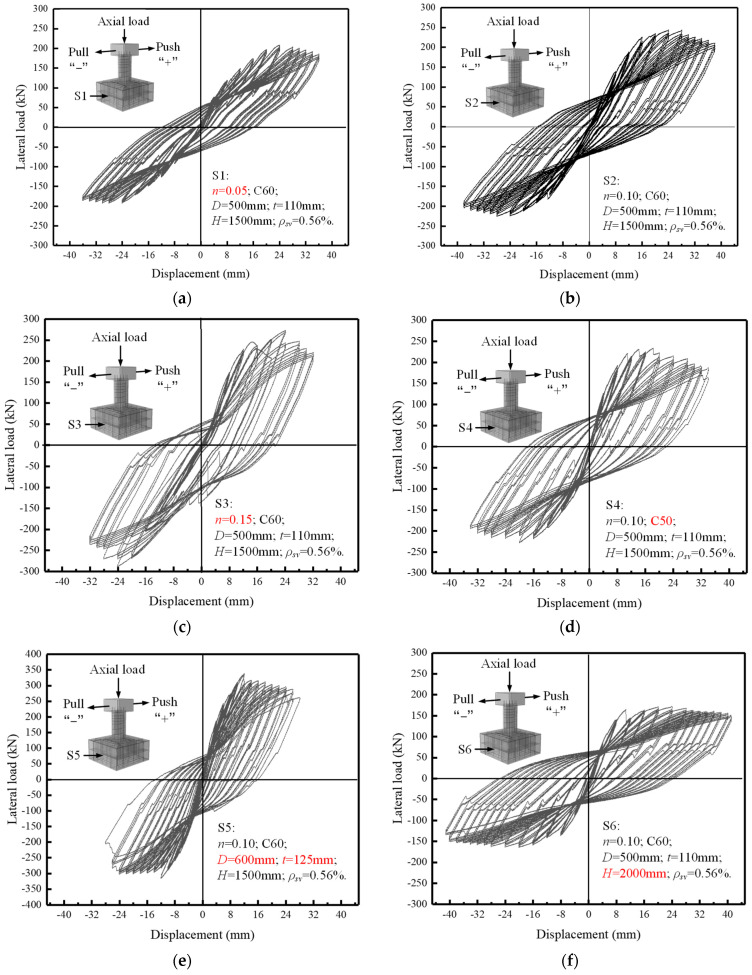

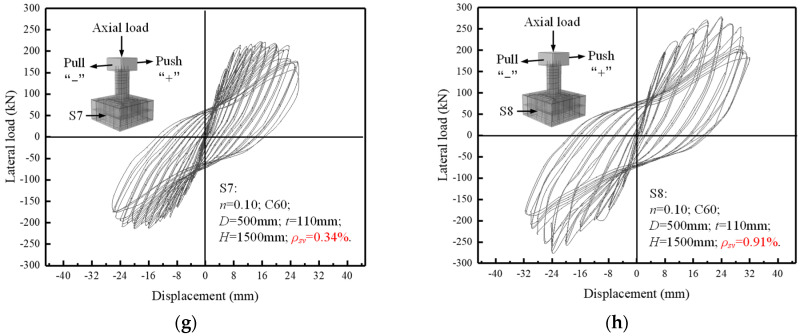
Hysteresis curves of the specimens, (**a**) S1, (**b**) S2, (**c**) S3, (**d**) S4, (**e**) S5, (**f**) S6, (**g**) S7, (**h**) S8.

Figure 7 shows the transverse load–displacement hysteresis curves of eight specimens in the loading process. These curves show the relationship between transverse load and displacement of the specimens’ cap beams. In general, at the initial loading stage of the eight specimens, before the internal longitudinal reinforcement yielded, the curves of the specimens were basically linear, reflecting the elastic state of the piers. With the increase in transverse displacement, the hysteresis loops became plumper due to the development of piers’ cracks and the slippage between concrete and reinforced bars. After the specimen experienced the peak load, the bearing capacity of the specimen decreased gradually with the increase in lateral displacement until the specimen was damaged.

In the group of axial compression ratio variables (S1, S2, and S3), the shape of the hysteretic curves of the three specimens was basically similar. However, under the same loading displacement level, the specimens with a larger axial compression ratio showed relatively higher transversal bearing capacity in the early loading stage, and the peak load of the specimens were larger. However, the bearing capacity of specimen S3 decreased significantly in the later stage, indicating that the axial compression ratio can improve the lateral bearing capacity of specimens within a certain range. However, the too-high axial compression ratio may cause insufficient ductility in the bearing capacity decline stage. This is because in the later period of larger lateral displacement, a larger axial force will produce a larger lateral bending moment for the piers. It is this effect that causes the specimen’s strength failure earlier in the later stage. Compared with specimen S2, specimen S4 showed a higher bearing capacity at the same initial displacement level, possibly due to manufacturing or test errors. However, from comparing the shape of the hysteretic curves of the two specimens, it could be seen that the reduction of concrete strength grade will lead to a slight decrease in the shear capacity. Compared with specimen S2, specimen S5 showed greater lateral bearing capacity in the early loading stage, and the hysteresis loop was more concentrated. With the further increase in the loading displacement level, the hysteretic loop area began to be gradually plump. There was a more obvious phenomenon of hysteretic curve pinching, and the specimen reached the ultimate failure in advance. However, under the condition of the same displacement loading level in the early stage, the horizontal load of specimen S6 was smaller. With the increase in the loading displacement, the hysteresis loop gradually became plump. Even though the specimen had experienced the peak load, its hysteresis curve did not show apparent fluctuation, indicating that the increase in shear span ratio can improve specimens’ ductility and energy dissipation capacity. In the group of stirrup ratio variables (S7, S2, and S8), the shape of the hysteretic curve of the specimen S7 was far less plump than that of S2, and the curve had apparent downward fluctuation at the declining stage of bearing capacity. While the specimen S8, with a higher stirrup ratio, maintained a higher lateral bearing capacity after entering the plastic stage, and the shape of the hysteresis loop was plumper than that of S2. Moreover, the hysteretic curve of specimen S8 was relatively stable after entering the bearing capacity decline stage, which reflected that the specimen had good ductility and dissipation capacity. It can be seen that the increase in stirrup can contribute more ultimate bearing capacity and energy dissipation capacity to the specimen.

### 3.3. Skeleton Curve and Ductility

**Figure 8:**
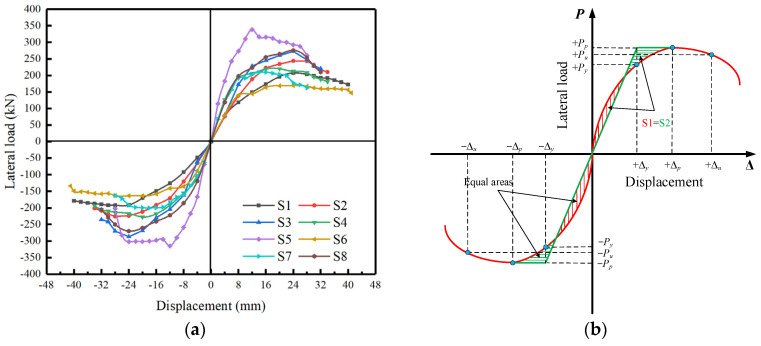
Skeleton curves of specimens and determination of ductility index, (**a**) Skeleton curves of specimens (**b**) The energy method.

The comparison of skeleton curves obtained from the hysteresis loops was shown in Figure 8a, and it could be seen that all skeleton curves were roughly similar in shape. The failure mode of each specimen can be divided into three stages according to its skeleton curve: the elastic stage, the strengthening stage, and the bearing capacity degradation stage. As can be seen from the skeleton curves of specimens S1~S3, the axial compression ratio had little influence on the piers at the elastic stage. The skeleton curves of the three specimens almost coincide in the early stage of displacement loading. However, with the increase in loading displacement, the transverse load, including the yield load and peak load, increased with the growth of the axial compression ratio under the same level of displacement, which can be considered that the existence of axial pressure would delay the time of the concrete crack and increase the yield load of the specimen. However, after entering the stage of bearing capacity degradation, the larger the axial compression ratio was, the faster the bearing capacity of the specimen decreased, and the specimen was damaged in advance. 

Moreover, from the perspective of specimen S4, the grade change in concrete strength did not lead to a significant influence. The specimen S4 showed a larger transverse bearing capacity in the elastic stage but entered the strengthening stage earlier. This phenomenon can be considered as the decrease in the concrete grade will make the rebars come into effect relatively earlier, leading to the improvement of the bearing capacity of the specimen in the early stage. However, finally, the peak and ultimate load of specimen S4 were slightly lower than specimen S2. As for specimen S5, it can be found that the increase in the pier body’s diameter offered the specimen a larger transverse bearing capacity under the same loading displacement level compared to specimen S2. However, the increase in diameter sharply shortened the displacement storage of the specimen in the strengthening stage. It made the peak load appear earlier, leading to a significant decrease in the specimen’s bearing capacity before the failure. As can be seen from the skeleton curve of specimen S6, the lateral bearing capacity of the specimen at the same loading displacement level decreased with the increase in the shear-span ratio while the overall curve showed a more gradual trend of uplift and decline at different stages. In addition, the skeleton curve of specimen S7 with the lowest stirrup ratio was lower than that of S8 on the whole, indicating that the transverse bearing capacity of specimens would generally increase with the increase in stirrup ratio before entering the degradation stage. While it can be seen that specimen S8, with the highest stirrup ratio, showed a rapid decline in the bearing capacity degradation stage, it may be considered that there should be a reasonable range of value for the stirrup ratio to improve the bearing capacity and ductility of the specimen.

Since there is no obvious yield displacement point in the skeleton curves, the energy method [30] was used to determine each specimen’s yield load and corresponding displacement, as shown in Figure 8b. The ultimate load was defined as 85% of the peak load [31], and the $\mu_{\Delta }$ was used to evaluate the ductility of specimens. The specific indicators are summarized in Table 4. As can be seen from Table 4, the characteristic loads of specimens S1, S2, and S3 were positively related to the axial compression ratio, and the characteristic displacements and displacement ductility coefficients were negatively related to it. Therefore, it can be concluded that the increase in axial compression ratio in a specific range is beneficial to the shear capacity of the specimens but has a negative impact on the ductility of the specimens. Every 5% increase in axial compression ratio contributes about 10–20% increase to the bearing capacity of the specimen, but the decrease in the ductility coefficient is not obvious. The characteristic load and displacement values of specimen S4 were slightly lower (nearly 10%) than those of specimen S2, indicating that the improvement of concrete strength grade can promote the shear capacity of the specimen. As for the influence of the shear-span ratio on specimens, the shear-bearing capacity of specimens at different stages was negatively correlated with the shear-span ratio. With the decrease in shear-span ratio, the bearing capacity of specimens in this test is increased by more than 30%. Both the increase in the shear-span ratio due to the height of the pier (S5) and the decrease in shear-span ratio due to the increase in pier diameter (S6) can improve the displacement ductility of specimens compared to the control specimen S2. Moreover, in terms of the stirrup ratio, it is evident that with the increase in the stirrup ratio, the shear bearing capacity (an increase of about 15%) and displacement ductility of specimens were significantly improved.

### 3.4. Degradation of Strength and Stiffness

According to the skeleton curves of each specimen, the degradation characteristics of stiffness and strength of each specimen were calculated to characterize the seismic performance of the specimen. The strength degradation coefficient of specimens was calculated as follows:

(1) $$\lambda_{i}=\frac{P_{i}}{P_{\max}}$$

where $\lambda_{i}$ is the strength degradation factor of the *i*th loading cycle, *P_i_* is the peak load in the *i*th loading cycle, and *P_max_* is the peak load in the skeleton curve of the specimen. 

The secant stiffness of specimens was defined as the ratio of peak load to corresponding displacement in each loading cycle, and the stiffness degradation coefficient was calculated as follows:

(2) $$\lambda_{iK}=\frac{K_{i}}{K_{0}}$$

where $\lambda_{iK}$ is the stiffness degradation factor of the *i*th loading cycle, *K_i_* is the average secant stiffness of the *i*th loading cycle, and *K*_0_ is the initial secant stiffness of specimen. 

**Figure 9:**
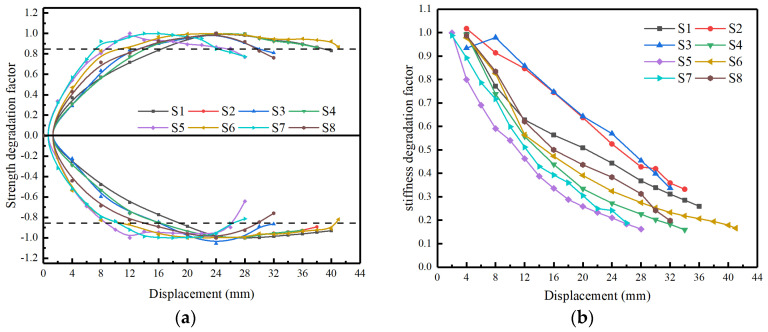
The strength and stiffness degradation curves of specimens, (**a**) Skeleton curves of specimens (**b**) The energy method.

The strength degradation curves of each specimen are shown in Figure 9a. The development trend of strength degradation curves of all specimens is basically similar. With the increase in lateral displacement, all specimens’ strength increased gradually, tended to a stable state, and finally failed. However, compared to the control group S2, specimen S5, with a larger pier diameter, and specimen S7, with a lower stirrup ratio, reached the peak strength relatively earlier. However, specimen S6, with a larger shear-span ratio, reached the ultimate strength at the latest, reflecting the good ductility of the specimen. As for the stiffness degradation curves of specimens in Figure 9b, it can be seen that all specimens’ stiffness decreased with the increase in lateral displacement and decreased rapidly in the early stage and then flattened out. This is because the cracks formed in the early loading stage weaken the specimen section’s effective area. After entering the bearing capacity degradation stage, there are generally no new cracks appearing. In addition, a larger axial compression ratio can inhibit the development of cracks in the early stage, resulting in higher initial stiffness of specimens. Therefore, specimens with relatively small axial compression tend to show more significant stiffness degradation in the early stage, and the increase in the stirrup ratio can alleviate the degradation degree of specimens to a certain extent.

### 3.5. Energy Dissipation and Viscous Damping

**Figure 10:**
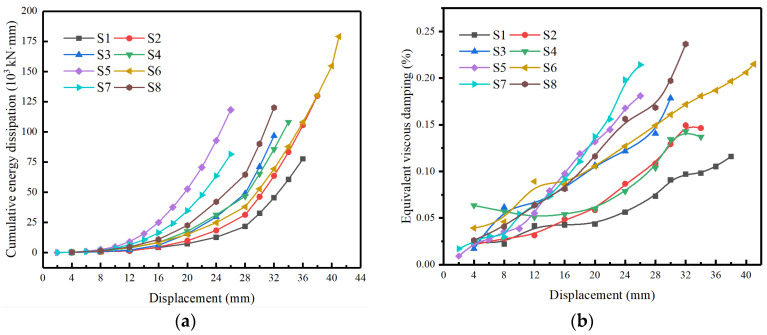
The energy dissipation curves of specimens, (**a**) The cumulative energy dissipation curves (**b**) The equivalent viscous damping curves.

In addition to the degradation of the strength and stiffness of the specimens, the energy dissipation capacity is also one of the significant indexes reflecting the seismic performance of the structure. In this paper, the area enclosed by the hysteresis curve was defined as the energy dissipation energy of the structure [32], which was used to reflect the energy dissipation capacity of the specimens under the inelastic hysteresis behavior. All specimens’ cumulative energy dissipation curves are shown in Figure 10a. Obviously, the total energy dissipation of S2 and S3 was significantly increased (about 50% and 20%) compared with S1, but the total energy dissipation of S3 was decreased compared with S2. Therefore, it can be inferred that the increase in axial compression ratio within a certain range can increase the energy dissipation of specimens, but too large an axial compression ratio would easily lead to early failure of specimens. The total energy dissipation of specimen S4 with lower concrete grade was basically consistent with that of specimen S2, indicating that this factor has no significant effect on the total energy dissipation of specimens. The energy dissipation of specimen S5 with a relatively more minor (under the influence of diameter) shear span was significantly higher than that of specimen S2 at the same displacement level, but the cumulative energy dissipation of specimen S5 did not change much due to the early failure of the specimen. At the same time, the cumulative energy consumption of S6 with a relatively larger shear span (under the influence of height) was significantly increased (about 110%) compared to S2. In addition, by comparing specimens S2, S7, and S8, it can be found that the cumulative energy consumption of specimens was positively related to the stirrup ratio, indicating that the increase in stirrup can significantly improve the energy dissipation characteristics of the specimens. 

In addition to reflecting the energy dissipation capacity of structures using the area of hysteretic curves, the equivalent viscous damping ratio is another energy dissipation evaluation index in seismic design. In this paper, the definition of equivalent viscous damping ratio in Clough and Penzien [33] was adopted as follows:

(3) $$\xi_{eqi}=\frac{1}{2\pi }\cdot \frac{E_{di}}{V_{mi}\Delta_{mi}}$$

where $\xi_{eqi}$ is the equivalent damping ratio of the *i*th cycle, *E_di_* is the area of the *i*th cycle hysteresis loop, and *V_mi_* and Δ*mi* are the average values of positive and negative peak load and corresponding displacements of the *i*th cycle, respectively.

Figure 10b shows the relation between the equivalent viscous damping ratio and the lateral displacement of each specimen. It can be seen that the equivalent viscous damping ratio of all specimens increased with the increase in lateral displacement, and changed faster at larger displacement levels. After entering the yield stage, the specimens with higher axial compression ratio showed bigger equivalent viscous damping ratio under the same loading displacement, and after entering the late loading stage, the curve of specimen S4 was basically consistent with that of specimen S2. In addition, the equivalent viscous damping ratio of S5 and S6 at the same displacement stage was bigger than that of S2. However, the specimen S7 with lower stirrup ratio reflected a higher equivalent viscous damping ratio probably due to the test errors, and the other rules reflected by the equivalent viscous damping ratio was consistent with that of cumulative energy dissipation.

## 4. Derivation of Shear Strength Model

### 4.1. Existing Shear Strength Models

Calculating the shear capacity of the reinforced concrete pier in the plastic hinge area is of great significance for seismic design and safety evaluation of engineering structures. At present, many shear strength models suitable for reinforced concrete solid piers have been proposed, and the prediction trend of shear strength of piers under the influence of different parameters has been verified by partial test data, but its applicability for circular hollow piers is still not explicit. Therefore, this study selected five existing shear capacity models [34,35,36,37,38] that considered different influencing factors or adopted different expression forms as the comparative group. The expressions of each shear capacity model and the main factors to be considered are shown in Table 5.

According to the expression forms of formulas in Table 1, only Priestley’s model divided the shear bearing capacity of piers into three parts: the bearing capacity *V_s_* provided by the stirrups, the bearing capacity *V_c_* provided by the concrete, and the bearing capacity *V_p_* contributed by the axial force. The other shear bearing capacity formulas are composed of the bearing capacity *V_c_* provided by concrete and the bearing capacity *V_s_* provided by the stirrups. Furthermore, in terms of the factors considered in the formula, both Priestley’s formula and Aschheim’s formula, considered the influence of the axial force and displacement ductility on the shear strength of specimens. On this basis, Sezen’s formula reflected the influence of shear span on shear capacity. In addition, the ACI 318-14 formula only considered the influence of axial force on the shear strength of specimens. In contrast, as for the influencing parameters considered by the other models, none of them were reflected in the shear capacity formula in the CHN-08 specification. Considering that all specimens studied in this paper are circular hollow piers, the forms of all model formulas in Table 5 have been unified on the original basis to facilitate later calculation and verification.

### 4.2. Proposed Shear Strength Model

According to the experimental results and existing literature, it can be seen that the shear-bearing capacity of the reinforced concrete pier is affected by the axial compression ratio, shear-span ratio, and stirrup ratio. Existing shear-bearing capacity models usually incorporate several influencing factors into the calculation formula. However, there are few shear-bearing capacity models for piers under the combined influence of axial compression ratio, shear-span ratio, and displacement ductility coefficient, and their applicability to the circular hollow piers remains to be verified. Therefore, considering that the shear bearing capacity of circular hollow piers increases with the increase in axial compression ratio, decreases with the increase in shear span ratio, and decreases with the increase in displacement ductility coefficient, the following shear bearing capacity formula was proposed:

(4) $$V_{cal}=V_{c}+V_{s}=\frac{an+b\mu_{\Delta }{}^{2}+c}{\lambda^{d}}\sqrt{f_{c}}A_{e}+\frac{\pi }{2}\frac{A_{s}{}_{v}f_{yv}D’}{s}\cot\theta$$

where *a*, *b*, *c,* and *d* represent the fitting parameters related to the bearing capacity factor, and the other symbols represent the same meanings as Table 6.

**Figure 11:**
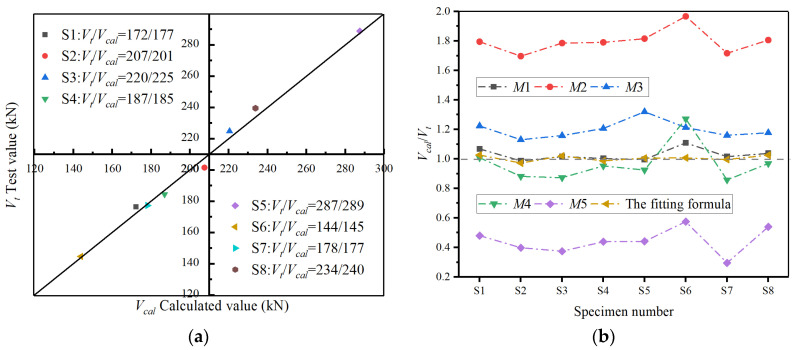
The comparison and verification of models, (**a**) The comparison between *V_t_* and *V_cal_* (**b**) The verification of several models.

In this formula, the shear capacity *V_cal_* of the circular hollow pier is composed of two parts: the shear capacity *V_c_* provided by the concrete and the shear force *V_s_* provided by the stirrup inside the pier. The contribution of the axial force on the specimen in the vertical direction to its shear strength was included in the *V_c_* part by introducing the parameter of axial compression ratio *n* while the *V_s_* part was calculated using the existing traditional truss model [39]. Moreover, considering that the specimens contain inner and outer stirrups with different configuration parameters, the outer stirrup contribution part *V_s_*_1_ and the inner stirrup contribution part *V_s_*_2_ were calculated separately and calculated to the *V_s_* part. The test shear capacity of the eight specimens and the specific parameters of the bearing capacity formula is shown in Table 6. Furthermore, the nonlinear fitting of the proposed shear capacity formula was carried out according to the relevant data. According to the fitting results, the fitting coefficients were *a* = 0.893, *b* = −0.026, *c* = 0.317, *d* = 0.205, and the correlation coefficient *R*^2^ = 0.953. Figure 11a shows the comparison between the calculated value obtained by the fitting formula and the test value, indicating that the fitting effect is good.

### 4.3. Comparison of Existing Shear Models’ Applicability

Considering that the applicability of the existing models to predict the shear capacity of the plastic hinge zone of circular hollow piers is not specific, the key geometric and loading parameters of eight piers in the test were substituted into different shear capacity models, and the shear capacity values calculated by different models were compared and analyzed with the test values. Table 5 contains the shear-bearing capacity models that need to be compared in this study, and Table 6 contains all the specimen parameters that need to be used in the model calculation. The ratio changes of calculated value *V_c_* and test value *V_t_* obtained with different model formulas of eight specimens are plotted in Figure 11b.

As can be seen from Figure 11b, the variation trend of the ratio of *V_c_* to *V_t_* of all models is basically the same, and the variation amplitude of some specimens is slightly different, which indicates that these formulas have a similar understanding of the shear mechanism of specimens. In addition, the formulas of Priestley and Sezen overestimated the shear capacity of prefabricated circular hollow piers, which made the seismic design of the hollow piers unsafe. In contrast, the CHN-08 formula underestimated the shear capacity of the plastic hinge area of piers, which is also not suitable for predicting the shear capacity of circular hollow piers. The average error between the calculated value and the test value obtained by Aschheim and ACI 318-14 formulas is basically controlled within 5%. However, the CHN-08 formula significantly overestimated the shear capacity of specimen S6 with a relatively larger shear span, which may be caused by the fact that this model did not consider that the increase in the shear-span ratio would inhibit the shear capacity of specimens. In contrast, Aschheim’s formula has better applicability in predicting the shear capacity of the plastic hinge area of prefabricated circular hollow piers within a certain ductility range (2 < $\mu_{\Delta }$ < 4). In practical engineering, full-size structures often show greater ductility than test specimens. Therefore, the formula of shear capacity proposed in this paper can be used in the actual design of prefabricated structure. At the same time, the design parameters of the structure can be also substituted into the formulas of Aschheim and ACI 318-14 to predict the pier’s bearing capacity for further comparison and verification.

## 5. Conclusions

In this paper, the seismic performance of prefabricated circular hollow piers with PVA concrete is studied by a quasi-static test. The failure process of specimens, transverse load-displacement hysteresis curves, skeleton curves, stiffness and strength degradation, and energy dissipation performance under different influence parameters were analyzed. Based on the test and analysis results, the shear capacity model of prefabricated circular hollow piers was fitted and verified. The specific conclusions are as follows:

(1) Excessive axial compression ratio (>15%) and stirrup ratio (>0.91%) will lead to a more significant spalling phenomenon when the specimen is damaged. The use of PVA concrete can produce finer cracks during the failure of the specimen, thus effectively improving the ductility and apparent failure morphology of the specimen.
(2) The bearing capacity and energy dissipation capacity of specimens are positively correlated with the axial compression ratio (5–15%). However, the excessive axial compression ratio will lead to insufficient ductility. The shear strength of specimens is negatively correlated with the shear-span ratio and positively correlated with the stirrup ratio, and the increase in the stirrup ratio and shear-span ratio caused by the change in pile height can effectively improve the ductility of specimens.
(3) In a certain range, the increase in axial compression ratio, stirrup ratio, and shear-span ratio caused by the change in pier height can significantly improve the energy dissipation characteristics of specimens, and the increase in the axial compression ratio will lead to a less apparent degradation of strength in the early stage and more significant degradation of strength in the later loading stage.
(4) The shear bearing capacity formula of plastic hinge area fitted in this study can effectively predict the shear bearing capacity of the circular hollow pier. Among the existing shear bearing capacity models, the formulas of Priestley and Sezen overestimate the shear capacity of circular hollow piers while the Aschheim and ACI 318-14 formulas have good applicability for predicting the shear capacity of such specimens in the plastic hinge area.

This paper explores the effects of axial compression ratio, concrete grade, shear-span ratio, and stirrup ratio on the seismic performance of prefabricated circular hollow piers. Based on this, the paper verifies and deduces the shear capacity model suitable for this kind of structure. It has a certain significance for the construction and design of prefabricated piers in practical engineering.

## Figures and Tables

**Table 1:** Main design parameters of the specimens.

Specimens	D (mm)	H (mm)	t (mm)	n	Pva Concrete	Longitudinal Rebar		Stirrup Rebar				
					Grade	Outer/Inner Layout	ρ_t_ (%)	ρ_sv_ (%)	d_s_ (mm)	R/R′ (mm)	L/L′ (mm)	s/s′ (mm)
S1	500	1500	110	0.05	C60	13φ12/6	1.36	0.56	6.0	220/160	360/720	90/180
S2	500	1500	110	0.10	C60	13φ12/6	1.36	0.56	6.0	220/160	360/720	90/180
S3	500	1500	110	0.15	C60	13φ12/6	1.36	0.56	6.0	220/160	360/720	90/180
S4	500	1500	110	0.10	C50	13φ12/6	1.36	0.56	6.0	220/160	360/720	90/180
S5	600	1500	125	0.10	C60	13φ13.5/8	1.35	0.56	6.0	270/195	350/700	70/140
S6	500	2000	110	0.10	C60	13φ12/6	1.36	0.56	6.0	220/160	360/1170	90/180
S7	500	1500	110	0.10	C60	13φ12/6	1.36	0.34	5.0	220/160	400/600	100/200
S8	500	1500	110	0.10	C60	13φ12/6	1.36	0.91	6.5	220/160	320/780	80/130

*D*: the outer diameter of specimens; *H*: the height of piers; *t*: the wall thickness of piers; *n*: the axial compression ratio; *ρ_t_*: the longitudinal reinforcement ratio; *ρ_sv_*: the stirrup ratio; *d_s_*: the diameter of stirrups; *R/R’*: the outer/inner stirrup’s spiral diameter; *L*: the length of the densification area scope of stirrups; *s*: the stirrup spacing of the densification area scope.

**Table 2:** Concrete mix of the PVA fiber concrete.

Grade	Cement (kg/m^3^)	Water (kg/m^3^)	Slag Powder (kg/m^3^)	Silicon Powder (kg/m^3^)	Water Reducer (kg/m^3^)	Quartz Sand (kg/m^3^)	PVA Fiber (%)
C50	406	150.0	100	18.4	7.5	632.4	0.5
C60	410	151.2	102	20.5	7.5	630.8	0.5

**Table 3:** The fundamental mechanical properties of masonry materials.

Location of Materials	Pier Body		Foundation/Bent Cap (The Post-Cast Part)	Contact Surface	Other Pre-Cast Parts
Material type	C50 (PVA)	C60 (PVA)	C50 (grouted material)	epoxy mortar	C60 (NC)
Compressive strength (MPa)	52.6	63.8	50.8	54.4 (28 h) 55.7 (28 d)	61.2

**Table 4:** The characteristic indexes of specimens during the loading process.

Specimens	*P_y_* (kN)	Δ*_y_* (mm)	*P_p_* (kN)	Δ*_y_* (mm)	*P_u_* (kN)	Δ*_u_* (mm)	$\mu_{\Delta }$
S1	190.00	17.4	207.66	24.6	172.13	39.6	2.28
S2	226.01	15.5	243.75	24.1	207.48	34.0	2.21
S3	235.27	13.7	272.40	23.2	220.42	29.8	2.17
S4	210.73	13.4	219.94	20.0	186.95	32.3	2.42
S5	329.75	11.4	338.23	12.5	287.47	26.6	2.33
S6	143.64	13.7	169.17	24.5	143.79	41.0	3.01
S7	192.02	11.5	209.62	14.5	178.18	24.2	2.10
S8	237.38	13.2	275.80	24.0	233.75	30.4	2.31

Δ*_y_*: the yield displacement; Δ*_y_*: the peak displacement; Δ*_u_*: the ultimate displacement; P*_y_*: the yield load corresponding to Δ*_y_*; P*_p_*: the peak load corresponding to Δ*_p_*; P*_u_*: the ultimate load corresponding to Δ*_u_*; $\mu_{\Delta }$: the ratio of Δ*_u_* to Δ*_y_*.

**Table 5:** The main existing models of shear capacity.

Formula Number	Source of Formula	*V* = *V_c_* + (*V_p_*) + *V_s_*	*k*
M1	Aschheim and Moehle	$0.3\times (k+\frac{N}{13.8A_{g}})\sqrt{f_{c}}A_{e}+\frac{\pi }{2}\frac{A_{s}{}_{v}f_{yv}d_{s}}{s}\cot30^{{}^{\circ}}$	$\frac{4-\mu_{\Delta }}{3},1<\mu_{\Delta }<4$
M2	Priestley et al.	$k\sqrt{f_{c}}A_{e}+0.85\times N\frac{D}{2L}+\frac{\pi }{2}\frac{A_{s}{}_{v}f_{yv}d_{s}}{s}\cot30^{{}^{\circ}}$	$-0.0835\mu_{\Delta }+0.417,2<\mu_{\Delta }<4$
M3	Sezen	$k(\frac{0.5\sqrt{f_{c}}}{H^{\prime }/D}\sqrt{1+\frac{N}{0.5\sqrt{f_{c}}A_{g}}}A_{e}+\frac{\pi }{2}\frac{A_{s}{}_{v}f_{yv}d_{s}}{s}\cot45^{{}^{\circ}})$	$1-\frac{\mu_{\Delta }-2}{4}\times 0.3,2<\mu_{\Delta }<4$
M4	CHN-08	$k\sqrt{f_{c}}A_{e}+\frac{\pi }{2}\frac{A_{s}{}_{v}f_{yv}d_{s}}{s}\cot45^{{}^{\circ}}$	0.023
M5	ACI 318-14	$k(1+\frac{N}{13.8A_{g}})\sqrt{f_{c}}A_{e}+\frac{\pi }{2}\frac{A_{s}{}_{v}f_{yv}d_{s}}{s}\cot45^{{}^{\circ}}$	0.167

*f_c_*: the design value of concrete compressive strength; *A_g_*: the cross section hair area of pier body; *A_e_*: the effective shear area of hollow pier section; *f_yv_*: the design value of stirrup tensile strength; *A_yv_*: the section area of stirrup in the same section; *H*′: the effective height of the pier from the top surface of the foundation to the loading midpoint of the cap beam; and the meanings of the remaining symbols are the same as those in Table 1.

**Table 6:** The main parameters of the model specimens.

Specimens	λ	*n*	$\mu_{\Delta }$	*f_c_* (N/mm^2^)	*A_e_* (mm^2^)	*A_sv_*_1and2_ (mm^2^)	*s*_1and2_ (mm)	${D^{\prime }}_{1}$(mm)	${D^{\prime }}_{2}$(mm)	*N* (kN)	*V_t_* (kN)
S1	2.4	0.05	2.28	27.5	107,819	28.27	180	464	320	185.31	172.13
S2	2.4	0.10	2.21	27.5	107,819	28.27	180	464	320	370.63	207.48
S3	2.4	0.15	2.17	27.5	107,819	28.27	180	464	320	555.94	220.42
S4	2.4	0.10	2.42	23.1	107,819	28.27	180	464	320	370.62	186.95
S5	2.0	0.10	2.33	27.5	149,226	28.27	140	564	390	512.96	287.47
S6	3.4	0.10	3.01	27.5	107,819	28.27	180	464	320	370.62	143.79
S7	2.4	0.10	2.10	27.5	107,819	19.64	220	464	320	370.62	178.18
S8	2.4	0.10	2.31	27.5	107,819	33.18	130	464	320	370.62	233.75

## Data Availability

The data used to support the findings of this study are available from the corresponding author upon request.
